# CD44 promotes multi-drug resistance by protecting P-glycoprotein from FBXO21-mediated ubiquitination

**DOI:** 10.18632/oncotarget.4763

**Published:** 2015-07-03

**Authors:** Abhilash K. Ravindranath, Swayamjot Kaur, Roman P. Wernyj, Muthu N. Kumaran, Karl E. Miletti-Gonzalez, Rigel Chan, Elaine Lim, Kiran Madura, Lorna Rodriguez-Rodriguez

**Affiliations:** ^1^ Rutgers Cancer Institute of New Jersey, New Brunswick, NJ, USA; ^2^ Rutgers Robert Wood Johnson Medical School, Piscataway, NJ, USA; ^3^ Department of Obstetrics and Gynecology, New Brunswick, Rutgers Cancer Institute of New Jersey, NJ, USA; ^4^ Present address: Delaware State University, Dover, DE, USA

**Keywords:** drug resistance, ubquitination

## Abstract

Here we demonstrate that a ubiquitin E3-ligase, FBXO21, targets the multidrug resistance transporter, ABCB1, also known as P-glycoprotein (P-gp), for proteasomal degradation. We also show that the Ser291-phosphorylated form of the multifunctional protein and stem cell marker, CD44, inhibits FBXO21-directed degradation of P-gp. Thus, CD44 increases P-gp mediated drug resistance and represents a potential therapeutic target in P-gp-positive cells.

## INTRODUCTION

The majority of cancer deaths are secondary to the development of metastases and tumor drug resistance although the biological link between these two phenotypes is unclear. In our previous work we showed that a membrane protein implicated in cancer metastases, CD44, activates the expression of a protein known to generate drug resistance, P-glycoprotein (P-gp), also known as the ABCB1 transporter [1]. Although the physical association of these 2 proteins was supported by their co-immunoprecipitation and co-localization within the cell membrane, and the induction of P-gp by CD44 overexpression was previously documented in our laboratory [1], the mechanism by which CD44 activates the drug resistance phenotype is still elusive.

The *CD44* gene contains 19 exons and is alternatively spliced, giving rise to many CD44 isoforms. When the central 10 exons are spliced out, the *CD44* “standard isoform” (CD44s) is expressed. CD44s, an ~85 kDa glycoprotein, is a receptor for hyaluronan, a major component of extracellular matrices. CD44 has been shown to be a useful tumor marker for disease progression and metastasis in certain types of cancers, and our laboratory, as well as others, has shown that CD44 is not usually expressed in the normal ovary although it is expressed in 40-60% of primary ovarian tumors [2–5]. Likewise, CD44 is not expressed in normal breast tissue but is expressed in 80% of metastatic breast cancer. Importantly, CD44 has been identified as a “stem cell” marker for both breast and ovarian cancer [6, 7].

P-gp, the product of the *MDR1* gene, is a 150-180 kDa, heavily glycosylated transmembrane ATP-dependent transporter known to pump cytotoxic drugs out of the cell, and its overexpression confers resistance to a variety of structurally diverse anticancer drugs such as vinblastine, doxorubicin and paclitaxel [8, 9]. Increased P-gp expression is observed in 60% of metastatic breast cancer and 30% of ovarian tumors [8], and is associated with poor outcome in cancer patients, presumably because it imparts resistance to cancer treatment [10, 11]. Despite efforts to develop drugs that interfere with the function of P-gp, the goal of restoring drug sensitivity in multidrug-resistant cells has not yet been clinically successful. Inhibition of the intrinsic mechanisms of the cell involved in controlling P-gp stability is an alternative approach to P-gp function interference. However, data from previous studies on the stability of P-gp are limited, and no evidence related to the targeting of P-gp for ubiquiination by an E3 ligase has been previously reported [12].

The purpose of this study was to elucidate a mechanism by which CD44 enhances P-gp-mediated drug resistance. We created a yeast two-hybrid system where transmembrane proteins can be expressed in the cell. Using this unique system, we were able to quickly evaluate the effect of CD44 mutations on P-gp function and expression. We used a high throughput siRNA ligase library screening method and a rapid *in vitro* test for P-gp function to select candidates for a P-gp-targeted E3 ligase. We present evidence to support FBXO21, an orphan E3 ligase, as the E3 ligase involved in the proteasome-mediated degradation of P-gp, and also elucidated a new mechanism for CD44 promotion of P-gp-directed drug resistance. Although proteasome inhibitors are currently being tested as anti-cancer therapy, major advances in treatment are more likely to be made through the targeting of specific ubiquitination factors such as E3 ligases. Furthermore, dual targeting of two cancer-related membrane proteins may allow for the preferential selection of cancer cells while sparing normal cells from drug toxicity.

## RESULTS

### CD44 interacts with P-glycoprotein in a yeast two hybrid system and increases P-gp induced drug resistance

**Figure 1:**
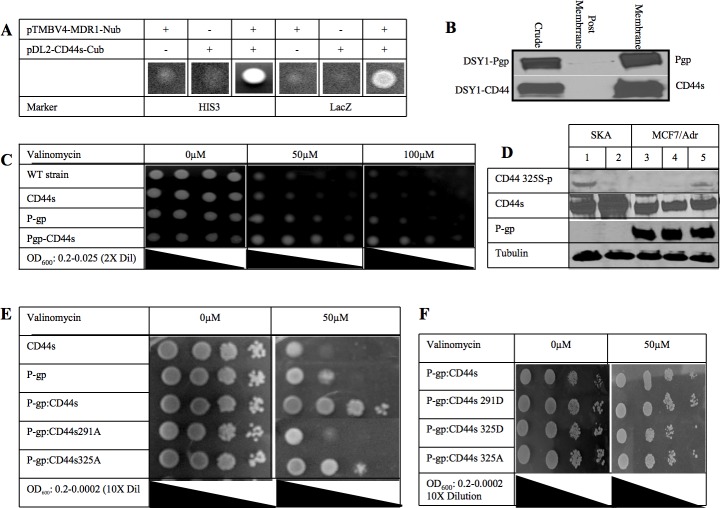
CD44 phosphorylation status affects P-glycoprotein mediated drug resistance **A.** A split ubiquitin yeast two-hybrid assay was used to detect protein-protein interactions between P-gp and CD44. Budding yeast DSY-1 cells expressing *MDR1* (in pTMBV4) and *CD44s* (in pDL2-X-Cub) as indicated, were grown on medium lacking leucine, tryptophan and histidine (HIS3) or containing X-Gal (LacZ), showing activation of the HIS3 and lacZ reporters respectively. When both *MDR1* and *CD44* genes were expressed yeast colonies grew in minimal media. **B.** Subcellular distribution of the expressed P-gp and CD44s. Membrane expression of both full length proteins is shown. **C.** DSY-1 cells expressing CD44, P-gp or both CD44 and P-gp along with untransformed wild type were grown overnight and serially diluted in liquid culture to an OD600 reading of 0.2, 0.1, 0.05 and 0.025 and spotted on a minimal synthetic plate with different concentrations of valinomycin as indicated. Colony spots were photographed as shown. **D.** Phosphorylation of CD44 at Ser 325 using an antibody specific for the CD44 Ser325 phosphorylated residue on Western blot analysis. Ovarian cancer cell line, SKA (Lanes 1 and 2) known to be P-gp(−)/CD44(+) and chemosensitive was compared with the MCF7/Adr cell line (Lanes 3-5) that is P-gp(+)/CD44(+). Cells were treated with PMA to induce PKC and hence phosphorylation of Ser291 of CD44 (Lane 2 and 4). Cells were treated with the PKC inhibitor bisindolylmaleimide to inhibit phosphorylation at Ser291 (Lane 5). Total amounts of CD44 and P-gp were determined by probing with the respective monoclonal antibodies. Anti-tubulin antibody was used as loading control. **E.** CD44 Ser to Ala mutations at either residue 291 or 325 were created and co-transformed with P-gp in the yeast two hybrid system. Serially 10-fold dilution cultures grown overnight were spotted on a minimal synthetic plate with or without 50μM valinomycin. Plates were incubated at 30°C for 3 days. Colony spots were photographed. **F.** CD44 Ser to Asp mutations at either residue 291 or 325 were created and co-transformed with P-gp in the yeast two hybrid system. Serially 10-fold dilution cultures grown overnight were spotted on a minimal synthetic plate with or without 50μM valinomycin. Plates were incubated at 30°C for 3 days. Colony spots were photographed.

To characterize the physical relationship between CD44 and P-gp, we took advantage of a split ubiquitin yeast two hybrid system that has the ability to screen for interactions among transmembrane proteins [13]. In this system, the *MDR1* gene was fused with the N-terminal half of ubiquitin and *CD44* was fused with the C-terminal half of ubiquitin which is linked to the artificial transcription factor PLV. Next, both plasmids were transformed into the reporter yeast strain (DSY-1). A positive interaction between P-gp and CD44 would allow the split ubiquitin to interact and conform to the structure of native ubiquitin. The assembled ubiquitin is recognized by a carboxy-terminal hydrolase and cleaved, thereby liberating the transcription activator PLV. Subsequently, PLV enters the nucleus by diffusion and binds to the LexA binding sites, leading to the activation of *LacZ* and *HIS3* reporter genes and resulting in blue cells in the presence of X-Gal and growth of the cells on plates lacking histidine, leucine and tryptophan, respectively. When the co-transfection of *CD44* and *MDR1* was performed in this yeast system, we observed blue colonies growing in the essential minimal media, indicating that the two proteins (ie, CD44 and P-gp) are close to each other at the cell membrane (Figure 1A). We corroborated the finding that both proteins are expressed at the cell membrane by subcellular fractionation and Western blot (Figure 1B).

We also tested whether the CD44/P-gp protein-protein interaction has an effect on drug resistance in yeast. Valinomycin is an ionophoric antibiotic that has the ability to cross the yeast cell wall and also has been shown to be a P-gp substrate [14]. Serially diluted cells were spotted on one plate without vancomycin and two plates containing different concentrations of valinomycin (Figure 1C). Valinomycin was found to be toxic to the wild type and the CD44 transformant strains. In contrast, the P-gp transformant cells were resistant to valinomycin, confirming that P-gp confers valinomycin resistance to yeast. Remarkably, the co-expression of CD44 with P-gp enhanced valinomycin resistance four-fold over the P-gp alone transformant yeast cells, while the presence of CD44 alone did not confer any drug resistance, indicating that the interaction of CD44 with P-gp, and not CD44 by itself, was conferring an increased drug resistance phenotype to the yeast strain.

### P-glycoprotein mediated drug resistance is dependent on the CD44 phosphorylation state

In resting cells, CD44 is constitutively phosphorylated at Ser325. After PKC is activated, a switch in phosphorylation occurs whereby Ser291 and Ser325 become phosphorylated and dephosophorylated, respectively [15]. The total amount of phosphorylation of CD44 remains the same, although only one of the two residues is phosphorylated at any given time. This phosphorylation switch was reported to result in the disengagement of ezrin and loss of cytoskeletal association [15, 16]. These findings prompted us to investigate whether the phosphorylation of CD44 was relevant to the increased drug resistance phenotype. Therefore, we examined the phosphorylation status of CD44 in P-gp-positive and P-gp-negative cell lines using SKA, an ovarian carcinoma cell line that expresses CD44 but not P-gp and MCF-7/Adr breast cancer cells that express both CD44s and P-gp, and a specific antibody to the CD44 Ser325 phophorylated residue. Figure 1D shows Western blots illustrating that CD44 is constitutively phosphorylated at Ser325 in SKA cells (Lane 1). When SKA cells were treated with protein kinase C activator (PMA), the dephosphorylation of Ser325 was apparent (Lane 2). Interestingly, in the MCF7/Adr cell line where both P-gp and CD44 are expressed, there was no constitutive phosphorylation at Ser325 (Lane 3) and no effect of PMA treatment (Lane 4). It is reasonable to assume that these cells constitutively express CD44 phosphorylated at Ser291. Because there are no antibodies specific to CD44 phosphorylated at Ser291, we inhibited PKC which is known to phosphorylate CD44 at this position. When MCF7/Adr cells were treated with a PKC inhibitor, CD44 Ser325 became phosphorylated, indicating that CD44 Ser291 is constitutively phorphorylated in MCF7/Adr cells (Lane 5). These experiments suggest that CD44 phosphorylation at Ser291 may be a key step in the binding of CD44 to P-gp and/or the CD44-related enhancement of the P-gp-induced drug resistance phenotype.

To determine whether changes in the phosphorylation of CD44 have an effect on CD44 binding to P-gp, CD44 Ser291 or Ser325 to alanine (Figure 1E) or aspartate (Figure 1F) point mutations were created and co-transformed with P-gp in yeast. Growth of these yeast strains in minimal media indicate that the binding of CD44 to P-gp was not inhibited by these point mutations (Figure 1E). However, when the functional role of P-gp in these strains was tested, we found that the CD44-Ser325Ala mutant showed a level of valinomycin resistance similar to that of wild type CD44 whereas the CD44-Ser291Ala mutant reversed the valinomycin resistance with the cells becoming as sensitive to the drug as the wild type strain in the presence of P-gp expression.

We further postulated that the substitution of serine by aspartate would mimic a “dominant ON” phosphorylation effect on the cells. Figure 1F illustrates that colonies co-expressing CD44 Ser291Asp with P-gp were as drug resistant as those colonies co-expressing wild type CD44 and P-gp. These results are consistent with the involvement of Ser291-phosphorylated CD44 in the drug resistant phenotype.

The phenotype exhibiting increased drug resistance that is observed when CD44 is expressed could be due to increased expression of P-gp either through transcriptional regulation or increased protein stability. However, in our yeast system the *MDR1* gene is not driven by its own promoter. Therefore, any transcriptional changes induced by CD44 are not detectable in this system. We corroborated this by qPCR. The *MDR1* transcript levels were the same in all of the strains, demonstrating that increased protein stability is a reasonable explanation for the increased drug resistance phenotype observed in yeast (Figure 1S).

### P-Glycoprotein protein stability is increased by CD44

**Figure 2:**
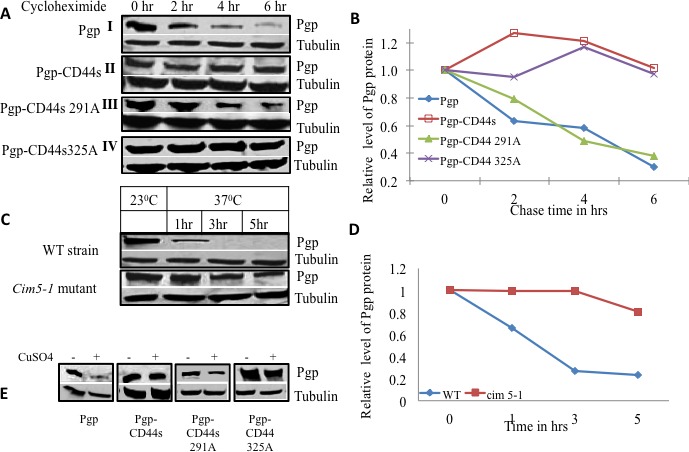
CD44 increases P-gp protein stability in yeast **A.** Yeast strains expressing either P-gp alone (**I**), P-gp and CD44 (**II**), P-gp and CD44 Ser291Ala mutant (**III**), or P-gp and CD44 Ser325Ala mutant (**IV**), were grown in overnight cultures, then treated with cycloheximide at a concentration of 10μg/ml for various times as indicated. Equal amounts of cell lysates from each treatment point were subjected to Western blot analysis using an anti-P-gp monoclonal antibody and an anti-alpha tubulin monoclonal antibody as loading control. **B.** The band intensities from blot A were quantified using densitometry. **C.** P-gp was expressed in the wild type yeast parent strain as well as in the conditional proteasome mutant *cim5-1* at the permissive temperature (23°C) and later at the non-permissive temperature (37°C) where the proteasome is nonfunctional in the *cim5-1* mutant. Aliquots were drawn at the respective time points as indicated and P-gp expression were evaluated by Western blot analysis using anti-P-gp along with alpha tubulin monoclonal antibodies **D.** The band intensities from blot C were quantified using densitometry. **E.** Yeast strains harboring P-gp with and without CD44 or its phosphorylation mutants were transformed with a myc-tagged ubiquitin plasmid with a CUP promoter. Cells were grown in the absence or presence of added CuSO4 as indicated. The expression of P-gp, and tubulin were studied by Western blot analysis using anti-P-gp, and anti-alpha tubulin monoclonal antibodies as labeled.

To test the effect of CD44 on P-gp stability, a cycloheximide chase on P-gp-expressing yeast strains with and without CD44s or CD44 phosphorylation mutant co-expression was performed (Figures 2A, 2B). After six hours of incubation in cycloheximide, cell lysates were prepared and separated by gel electrophoresis. Western blotting revealed that more than 50% of P-gp was degraded within four hours in the strain that expressed P-gp alone (Figure 2A, Block 1). On the other hand, the yeast strain that co-expressed P-gp and CD44 exhibited a longer P-gp half-life that exceeded 6 hours (Figure 2A Block II). We then tested P-gp stability when it was co-expressed with the CD44 Ser291Ala phosphorylation mutant. Interestingly, the stability of Pg-p was reverted back to that observed with the P-gp alone phenotype, indicating that the CD44 Ser291Ala mutant did not show any protective role on the half-life of P-gp (4 hrs. *vs*. 4.5 hrs for P-gp alone expressing cells (Figure 2A Block III and Figure 2B). On the other hand, the phenotype of the CD44 Ser325Ala mutant was similar to that of wild type CD44/P-gp transformant; it imparted the same increased half-life to P-gp as wild type CD44 (Figure 2A Block IV and Figure 2B). These experiments indicate that CD44 confers stability to P-gp or alternatively, protects P-gp from degradation when CD44 is phosphorylated at Ser291.

### P-glycoprotein is degraded through the proteasomal pathway

To investigate whether P-gp stability was regulated by 26S proteasome-mediated degradation, P-gp was overexpressed in yeast strains harboring conditional mutations in the 19S regulatory sub-unit of the 26S proteasome and in the wild type parent strain. The conditional alleles are in the gene encoding the *AAA^+^* ATPase Cim5. We measured the level of P-gp expression in the parent strain and in the heat sensitive *cim5-1* mutant at the permissive temperature (23°C) and then at the non-permissive temperature (37°C) at different points in time (Figure 2C). The level of P-gp expression at the permissive temperature was considered the basal level for both the mutant and parent strain. The proteasome machinery is fully functional at the permissive temperature, both in wild type and mutant strains, whereas the proteasome is impaired in the mutant strain only at the non-permissive temperature. At 37°C, P-gp was degraded significantly faster in the parent strain compared with the *cim5-1* mutant strain. Figure 2D shows a graphical representation of the P-gp turnover in the *cim5-1* mutant and the parent strain at the non-permissive temperature. More than 50% of the P-gp protein was degraded during the first 2 hrs at 37°C in the wild type strain. In contrast, in the *cim5-1* mutant strain, the majority of the P-gp accumulated even after 5 hrs at the non-permissive temperature where the proteasome is inactive (Figure 2D). These results reveal that P-gp is degraded by the proteasomal pathway; we therefore postulated that ubiquitin would be involved in the process.

To determine the role of ubiquitin in the degradation of P-gp, we used a functional c-myc epitope-tagged ubiquitin (myc-Ub). Yeast strains expressing P-gp alone, P-gp with either wild type CD44 or the phosphorylation defective CD44 mutants at Ser291 or Ser325 were transformed with YEp105 under the control of the *CUP1* promoter. Cells were grown in the presence or absence of CuSO4. Cell lysates were prepared and the levels of P-gp were examined by Western blot and densitometry. Figure 2E illustrates that the level of P-gp was significantly lower in the transformant that expressed P-gp alone after induction of ubiquitin activity with CuSO4. On the other hand, the P-gp level remained the same in the wild type CD44 strain before and after induction of the c-myc tagged ubiquitin. Importantly, the P-gp level in the yeast strains with the CD44 Ser291Ala mutant was decreased when ubiquitin was induced, whereas the amount of P-gp remained stable after the induction of ubiquitin in the CD44 Ser325Ala mutant strain, showing a similar degree of P-gp stability as observed when wild type CD44 was expressed.

### CD44 protects P-gp from ubiquitination in mammalian cells

**Figure 3:**
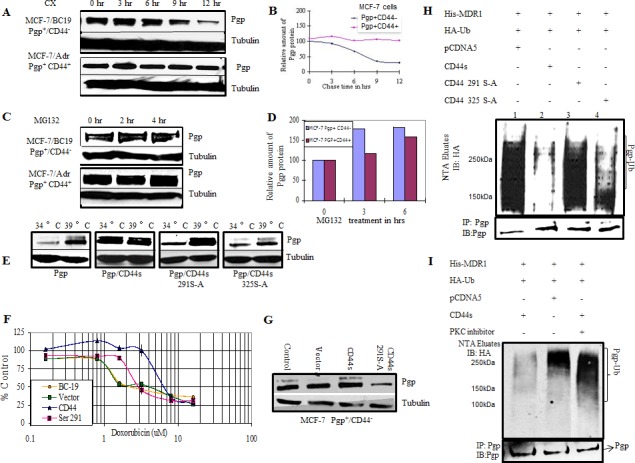
CD44 protects P-gp from ubiquitination **A.** MCF-7/BC19 and MCF-7/Adr cells were treated with 50μg/ml cycloheximide. Cells were harvested at the designated time points. Total cell lysates were prepared and subjected to Western blotting analysis to detect the amount of P-gp present on the cells. Tubulin was used as a loading control. **B.** The band intensities from blot A were quantified using densitometry. **C.** MCF-7/BC19 and MCF-7/Adr cells were treated with the proteasome inhibitor MG 132 to a final concentration of 10μM for 2 hr and 4 hr as indicated. Total cell lysates were prepared and subjected to Western blot analysis probed with anti-P-gp and anti-tubulin monoclonal antibodies. **D.** The band intensities from blot C were quantified using densitometry. **E.** CD44 and phosphorylation mutants at residue 291 or 325 were stably expressed in ts20 3T3 cells that contained a temperature-sensitive defect in the E1 ubiquitin activating enzyme. P-gp was then transiently transfected in these cell lines and initially cultured at the permissive temperature (34°C) before being cultured at the restrictive (39°C) temperature (ubiquitination defective) for 18 hours. Cell lysates were collected and probed with anti-P-gp and anti-tubulin monoclonal antibodies. **F.** MCF-7/BC19 cells were transfected with either empty vector, full length CD44s or the CD44 Ser291Ala mutant, and treated with varying concentrations of doxorubicin. The MTT assay was performed and the viability of each cell line was plotted as percent of control against the concentration of drug used. **G.** Aliquots from each transfectant used on panel F at time zero were taken to perform Western blots and determine P-gp expression. **H.** *In vivo* ubiquitination experiments were performed as follows: HEK293T cells were co-transfected with His-tagged MDR1, HA-tagged ubiquitin and either wild-type CD44, CD44 Ser291Ala mutant, CD44 Ser325Ala mutant or empty vector as indicated. His-tagged P-gp was purified on Ni-NTA resin under denaturing conditions; the eluates were separated using SDS PAGE. The presence of ubiquitinated P-gp was determined by immunoblotting with anti-HA antibody. The lower panel demonstrates co-immunoprecipitation with anti-P-gp antibodies to determine the P-gp level of each transfectant. **I.** *In vivo* ubiquitination experiments were performed in the presence or absence of a PKC inhibitor as indicated. HEK293T cells were transfected with the indicated plasmids. After 48 hrs, the cells were treated with bisindolylmaleimide, a PKC inhibitor, for 4 hrs. The ubiquitinated proteins were purified on Ni-NTA resin under denaturing conditions and immunoblotted with anti-HA antibody. The upper panel shows the ubiquitinated species (P-gp-Ub). The lower panel represents co-immunoprecipitation with anti-P-gp antibodies to determine the P-gp level of each transfectant.

To study whether the CD44-mediated protection of P-gp from ubiquitination is also present in mammalian cells, we tested the stability of P-gp using a cycloheximide chase analysis in a breast cancer cell line that expresses P-gp but not CD44 (MCF-7/BC19) and in a cell line that expresses both P-gp and CD44 (MCF-7/Adr). We performed Western blots of both cell lines after cycloheximide treatment at different time points. As observed in yeast, the results show that the degradation of P-gp was significantly delayed in the cell line that co-expresses CD44 and P-gp when compared with the MCF-7/BC19 cells that express P-gp but not CD44 (Figure 3A). Figure 3B shows a graphical representation of a densitometry analysis of the Western blot depicting that more than 50% of P-gp was degraded in 6.5 hrs in the P-gp(+)/CD44(−) cell line. On the other hand, when P-gp and CD44 were co-expressed, the amount of P-gp expressed remained stable even after 12 hrs of treatment with cycloheximide. Thus, the stability of P-gp is also critically dependent on CD44 expression in mammalian cells. We then tested for the role of proteasomal degradation of P-gp using the proteasome inhibitor MG132 to block ubiquitin-dependent degradation (Figure 3C and 3D). We found that when proteasome function was inhibited in MCF-7/BC19 cells, P-gp accumulated in a time dependent fashion. On the other hand, when P-gp was co-expressed with CD44 there was no significant change in the level of P-gp measured before and after proteasomal inhibition. These results suggest that CD44 promotes the stability of P-gp by inhibiting the proteasome-mediated degradation of P-gp.

We next sought to test the role of CD44 and its phosphorylation status on P-gp ubiquitination in mammalian cells. We used a temperature-sensitive ubiquitination mutant, ts20 3T3, derived from the mouse cell line BALB/c 3T3 [17]. Full length CD44 or either one of the CD44 phosphorylation deficient mutants Ser291Ala and Ser325Ala were transfected into the temperature sensitive cell line and stable transfectants were selected. *MDR1* was co-transfected transiently into the cell lines and assayed for accumulation of P-gp at the ubiquitination permissive temperature (34°C) and after a shift to the ubiquitination restrictive temperature (39°C) for 6 hrs. Figure 3E shows immunoblots probed with anti-P-gp antibodies to compare the level of P-gp expression among the different cells lines at 34°C and 39°C. We detected low levels of P-gp in cells expressing P-gp alone at the ubiquitination permissive temperature. When ubiquitination was inhibited by raising the temperature to 39°C, the P-gp band accumulated, indicating a role for ubiquitination in the P-gp stability of mammalian cells. On the other hand, the cell line that co-expressed CD44 and P-gp maintained the same level of P-gp expression whether ubiquitination was active or not, indicating that CD44 protected P-gp from ubiquitin-directed degradation. Cells co-expressing P-gp with CD44 Ser291Ala mutant protein showed accumulation of P-gp at 39°C, similar to the accumulation observed in the P-gp (+)/CD44 (−) cell line. This experiment demonstrates that the CD44-protective effect on P-gp degradation is absent in cells expressing the CD44 Ser291Ala mutant. On the other hand, cells expressing the CD44 Ser325Ala mutant showed an amount of protection against P-gp degradation similar to that rendered by wild type CD44. These results confirm our findings in the yeast system and further establish that CD44 protects P-gp from ubiquitin-targeted degradation. Furthermore, CD44 phosphorylation at Ser291 is demonstrated to be essential for this function. To further evaluate the role of CD44 on the P-gp-induced drug resistant phenotype, and to determine whether P-gp was functional, the cytotoxicity of doxorubicin, a known substrate of P-gp, was analyzed in breast cancer cells (Figure 3F). Cell viability was determined by MTT assays following treatment with doxorubicin. P-gp-positive cells transfected with empty vector were used as a control. Similar to the yeast system, CD44 induced a higher level of drug resistance that was reversed when the CD44 Ser291Ala mutation was introduced. Western blotting demonstrated increased expression of P-gp in CD44 transfectants compared with P-gp expression in CD44 Ser291Ala mutant cells and the empty vector transfectants (Figure 3G).

### P-gp is ubiquitinated *in vivo*

*In vivo* ubiquitination experiments were performed to verify ubiquitination of P-gp. A co-transfection of His-tagged MDR1, CD44 and HA-tagged ubiquitin in HEK293T cells followed by purification of ubiquitinated proteins on nickel resins, allowed detection of ubiquitinated P-gp *in vivo* (Figure 3H). Lane 1 reveals that P-gp is ubiquitinated *in vivo* and shows its basal level of ubiquitination. Lane 2 shows that the overall level of ubiquitinated P-gp was reduced by the expression of full-length wild type CD44 when compared with control CD44-negative cells in Lane 1. Interestingly, P-gp ubiquitination was noticeably increased when the CD44 Ser291Ala phosphorylation mutant was expressed (Lane 3). Conversely, when the Ser325Ala mutant was expressed, P-gp was again protected from ubiquitination at a level similar to the protection conferred by wild type CD44 (Lane 4).

To corroborate that phosphorylation at Ser291 is required for CD44-mediated protection of P-gp from ubiquitination, we inhibited Ser291 phosphorylation by using bisindolymaleimide, a PKC inhibitor. An additional *in vivo* ubiquitination experiment was done using HEK293T cells co-transfected with His-tagged P-gp, ubiquitin and CD44 constructs. The ubiquitination level of P-gp was detected by immunoblotting. Figure 3I shows that bisindolylmaleimide treatment inhibited the protective effect of CD44 on P-gp ubiquitination in the CD44 transfected group (Lane 3) when compared with the no treatment group. The overall P-gp ubiquitination level when CD44 was not expressed was similar to the P-gp ubiquitination level when CD44 was expressed after treatment with the PKC inhibitor. These results corroborate that the phosphorylation of CD44 at Ser291 is required for the CD44 protection of P-gp from ubiquitination.

### FBXO21 is an E3 ligase for P-gp

**Figure 4:**
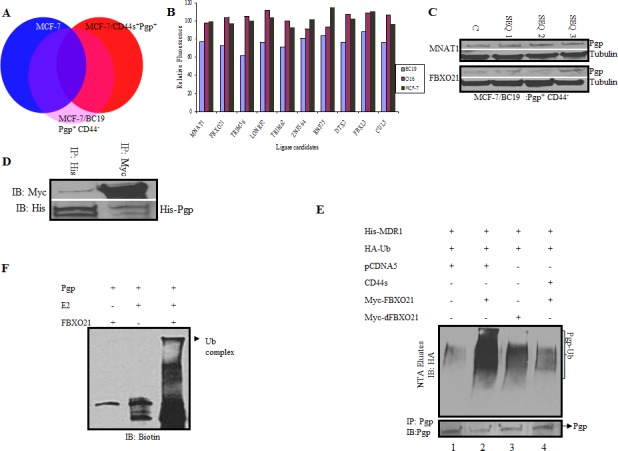
A genome wide siRNA ubiquitin ligase screen identifies FBXO21 as the E3 ligase targeting P-gp for ubiquitination **A.** Parental MCF-7 cells (P-gp(−)/CD44(−)), MCF-7/P-gp(+)/CD44(−), and MCF-7/P-gp(+)/CD44(+) cells were screened with an siRNA ubiquitin ligase library targeting 329 known and predicted ubiquitin ligases. A change in drug uptake ability between the different genotypes was measured using calcein AM, a fluorescent substrate for P-gp. Candidate genes directly affected by siRNAs only in MCF-7/P-gp(+) and not in MCF-7/P-gp(+)/CD44(+) and parental MCF7 (P-gp(−)/CD44(−) cells) with the highest p-value in three independent primary screens, were considered as potential hits and used for subsequent analysis (Labeled as pink on diagram). **B.** Graph comparing calcein uptake from different cell lines after siRNA treatment of the first 2 potential ligases chosen from the screen described in A. **C.** Cells treated with different sequences of siRNAs targeting the two top hits were further validated by qPCR for the respective genes. **D.** HEK 293 T cells were transfected with His-tagged P-gp and myc-tagged FBXO21. Cells were harvested and lysed after 48 hrs. Whole cell lysates were subjected to reciprocal immunoprecipitation and immunoblotted with respective antibodies. **E.** HEK293T cells were transfected with His-tagged P-gp, HA tagged ubiquitin, CD44, FBXO21 or dFBXO21. Cell lysates were separated and His-tagged P-gp was purified on Ni-NTA resin under denaturing conditions; the eluates were separated in SDS PAGE. The presence of ubiquitinated P-gp was determined by immunoblotting with anti-HA antibody. The ubiquitinated P-gp smear is indicated by a bracket. The lower panel represents co-immunoprecipitation of aliquots from the same cells with anti-P-gp antibodies to determine the P-gp level of each transfectant. **F.** *In vitro* ubiquitination studies were performed using recombinant P-gp and FBXO21 purified from Sf-9 insect cells along with E2 and biotinylated ubiquitin. The components of the reaction were separated by SDS PAGE and immunoblotted with streptavidin-conjugated antibody.

To identify an E3 ubiquitin ligase involved in the degradation of P-gp, we performed an RNAi library screen on cells expressing either P-gp alone, both P-gp and CD44 or neither P-gp nor CD44. A SMARTpool library of 329 siRNAs targeting known or predicted human E3 ubiquitin ligases was transfected into the above mentioned cell lines along with a nontargeting control siRNA. Fluorescent substrate accumulation assays to measure P-gp efflux function was performed on the siRNA-treated cells using calcein AM as substrate. This assay measures the amount of calcein AM that is recognized and exported by P-gp from the plasma membrane of cells into the outside media. Cells that do not express P-gp would accumulate more intracellular fluorescence substrate compared to the cells that express P-gp. We aimed to identify those siRNAs that specifically increased the function of P-gp only in the cells that were P-gp(+)/CD44(−) and not in the cells expressing both P-gp and CD44 or no P-gp (Figure 4A, labeled pink). Most of the siRNAs targeting genes in more than one group were eliminated from the final analysis because those genes would affect drug resistance independent of P-gp. After the analysis, a short list of 10 genes was identified that specifically increased the function of P-gp in P-gp(+)/CD44(−) cells and therefore had the potential to specifically inhibit the function of the P-gp ligase. The list of genes identified and the normalized values are given in Table S1. To validate possible E3 ligase candidates we tested for fluorescence uptake after transfecting P-gp(+)/CD44(−) cells with different E3 ligase siRNAs (Figure 4B). Our analysis depicted MNAT1 as the best candidate for the E3 ligase (Figure 4C); however, when tested, it did not show E3 ligase activity (data not shown). We next screened FBXO21 as a potential P-gp E3 ligase. We first determined whether FBXO21 would bind to P-gp using co-immunoprecipitation experiments. We performed reciprocal co-immunoprecipitation studies in which recombinant Myc-tagged FBXO21 and His-tagged P-gp were transiently expressed in HEK293T cells. Figure 4D illustrates that FBXO21 co-immunoprecipitated with P-gp.

*In vivo* ubiquitination studies were done by co-transfecting His-tagged P-gp with HA-tagged ubiquitin along with wild type and/or the dominant-negative FBXO21 mutant (dFBXO21) in HEK293T cells with or without CD44. Figure 4E shows that the level of ubiquitin-conjugated P-gp was noticeably increased when FBXO21 was overexpressed (Lane 2, upper panel) and the overall level of His-P-gp was concomitantly reduced (Lane 2, lower panel). As expected, the expression of dFBXO21 inhibited the activity of FBXO21 as evidenced by a decreased level of ubiquitination and increased expression of P-gp (Lane 3). Furthermore, CD44 protected P-gp from ubiquitination with FBXO21 as indicated by the less prominent an ubiquitination smear (Lane 4, upper panel).

**Figure 5:**
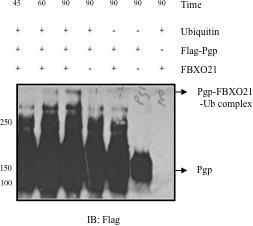
*In vitro* ubiquitination of P-gp (time course) Purified *in vitro* translated FLAG-tagged P-gp along with E2 (UbcH5c), E3 ligase FBXO21, Cul1, Skp1, Roc1 purified from Sf-9 insect cells were incubated *in vitro*. Reactions were started by adding ATP and stopped by adding sample buffer at different time points. Samples were analyzed by immunoblotting and anti-FLAG antibody.

Having established FBXO21-mediated ubiquitination of P-gp *in vivo*, we performed an *in vitro* reconstitution experiment. Affinity-purified FLAG-tagged P-gp, FBXO21, Skpl, Cul1 and Roc1 were pre-incubated together to allow for the assembly of the SCF-P-gp complex. Ubiquitination was then initiated by the addition of biotinylated-ubiquitin, E1, E2, PKC, and ATP. The reaction products were analyzed for a higher molecular weight smear characteristic of ubiquitinated proteins. Figure 4F shows that when all the components were present, a high molecular weight band was evident as a ubiquitinated complex. We then performed an *in vitro* reconstitution time course experiment where a FLAG-tagged P-gp was used as a substrate and mixed with purified E2, FBXO21-SCF-CUL1 and ubiquitin. The reaction was started by the addition of ATP and stopped by the addition of sample buffer at different time points. The ubiquitinated P-gp complexes were analyzed by Western blotting probing with anti-FLAG antibody. Figure 5 depicts a P-gp complex evident at 45 minutes that increases in intensity by 90 minutes. Exclusion of either ubiquitin or FBXO21 completely blocked the reaction. These observations provide definitive biochemical evidence that P-gp ubiquitination can be reconstituted *in vitro* and that FBXO21 is an E3 ubiquitin ligase for P-gp.

## DISCUSSION

Multidrug resistance, a major reason for failure of anti-cancer therapy, can be caused by the overexpression of P-gp, a member of the ABC family of transporters [18, 19]. Although *in vitro* inhibition of the P-gp drug transport pump causes cancer cells to become drug sensitive, clinical trials have failed to replicate the *in vitro* findings, mostly as a result of complications related to unanticipated toxicities [20]. In a previous study, we treated an ovarian cancer mouse xenograft with siRNA targeting CD44, and showed that the resulting reduction of CD44 expression in the tumor correlated with decreased expression of P-gp and increased drug cytotoxicity [21]. Our present work provides further insight into the regulation of P-gp expression by CD44 which is needed to more effectively design the next generation of compounds that will both overcome multidrug resistance in cancer cells and be less toxic to normal cells.

The ubiquitination and subsequent proteasomal processing of other integral membrane transporters such as the cystic fibrosis transmembrane conductance regulator (CFTR) has been previously reported [22–24]. In addition, Ohkawa and colleagues demonstrated that ubiquitinated P-gp accumulates after inhibition of calpain hydrolyisis [25]. Glycosylated, fully-mature P-gp is known to have a long half-life (14 hrs),[26–28] and P-gp ubiquitination of unglycosylated P-gp has been shown to be independent of P-gp phosphorylation [12]. However, the mechanisms governing the proteasomal degradation of mature P-gp have remained largely unknown. The data presented here provide molecular insight into the mechanism of P-gp degradation, a process that is almost completely impeded by the cellular expression of CD44. Furthermore, these results support the existence of a newly uncovered mechanism by which CD44 increases drug resistance in cancer cells.

In the present work, a yeast two-hybrid system was designed specifically for the study of membrane protein interactions. In this system, P-gp is expressed in a fully functional form as an integral protein capable of pumping out valinomycin, a P-gp substrate toxic to yeast cells; it also allows for the systematic investigation of the binding between P-gp and CD44 to determine the effects of this interaction on drug resistance. Our results reveal a previously unknown function for CD44 in the promotion of drug resistance through stabilization of P-gp and inhibition of its proteasome-targeted ubiquitination.

Misra *et.al.* reported that CD44 regulates phosphoinositide 3-kinase (PI3-kinase) activity, an enzyme involved in the stimulation of P-gp expression [29]. In addition, we previously showed that CD44 induced P-gp expression at the mRNA level [1]. Our new findings expand the current understanding of the regulation of cancer drug resistance by CD44, demonstrating the CD44-mediated inhibition of P-gp ubiquitination. Our work, together with the work of Misra and colleagues, demonstrates that P-gp expression in cancer cells is tightly controlled by CD44.

In this work, P-gp (ABCB1 transporter) is identified as a physiologic substrate of FBXO21, an orphan E3 ligase of the F-box family of ligases which are characterized as being without a recognizable protein interaction domain [30–32]. In addition, FBXO21 is shown to catalyze ubiquitination of P-gp, thereby targeting it for subsequent proteasomal degradation. Further, a phosphorylated form of CD44 is shown to be a strong inhibitor of FBXO21-directed ubiquitination of P-gp. Although phosphorylation of P-gp is not necessary for its ubiquitination, the degree of P-gp stability is critically dependent on the PKC-catalyzed phosphorylation of CD44 at its Ser291 residue. We also show here that mutations of CD44 at Ser291 are associated with increased cellular sensitivity to chemotherapy drugs as well as lower expression of P-gp. These results demonstrate that the protection of P-gp ubiquitination provided by CD44 is functional and biologically relevant. CD44 is constitutively phosphorylated at Ser325 and this form of the receptor binds to ezrin, the protein that links CD44 to the actin cytoskeleton. A concomitant dephosphorylation of Ser325 and phosphorylation of Ser291 ensues on PKC activation of CD44, resulting in the disengagement of ezrin and loss of cytoskeletal association [15]. Interestingly, P-gp also binds to ezrin and inhibition of ezrin synthesis restores drug sensitivity in P-gp-positive cells [33]. Whether ezrin needs to be disengaged from CD44 and subsequently bind to P-gp for CD44 to protect P-gp from ubiquitin-mediated degradation is yet to be determined.

In conclusion, this work elucidates a previously unknown mechanism of P-gp control by CD44 that could be exploited in the design of the next generation of P-gp antagonists. By targeting only those cells expressing both CD44 and P-gp, it may be possible to circumvent the drug toxicities previously encountered in clinical trials of agents targeting P-gp function alone. Using this approach, normal cells from the gastrointestinal tract and blood-brain barrier that constitutively overexpress P-gp would not be targeted and toxicities to these organs would be spared. We also speculate that by targeting P-gp-positive/CD44-positive cancer cells, we will be aiming at cancer stem cells that are slow growing and more difficult to kill with standard cytotoxic agents.

Finally, we can extrapolate our findings to other areas of biology and medicine, such as the use of protease inhibitors and cyclosporine in the setting of AIDS and transplantation, respectively [34, 35]. In those situations, targeting CD44 may also provide a less toxic approach than direct inhibiton of P-gp. [36, 37].

## MATERIALS AND METHODS

### Strains and growth conditions

*Saccharomyces cerevisiae* strains DSY-1 (MATa his3Z200 trp1-901, leu2-3, 112 ade2LYS2::(lexAop)4-HIS3 URA3::lexAop)8-lacZ GAL4), and CMY 791 (MATa ura 3-52, leu 2Z1, his-3-Z200, cim5-1) were used for the studies. Cells were grown in either YTDA or SD dropout medium at 30°C for the normal strains and 24°C for *cim5-1* temperature-sensitive strains [38]. The AAA+ ATPase Cim5 proteasome mutant yeast strain was provided by K. Madura [38]. C-myc tagged ubiquitin was expressed from the yeast-*Escherichia coli* plasmid, YEp105, under the control of *CUP1* promoter by the addition of copper sulfate (CuSO4) (at a final concentration of 100uM) for 30 minutes [39].

### Mammalian cell lines

The source and maintenance of the human breast cancer cell lines were previously described [1]. The human ovarian carcinoma cell line, SKA, was provided by Dr John Ludlow (University of Rochester, NY). They were maintained in DMEM (Life Technologies, Carlsbad, CA), supplemented with 10% FCS (Life Technologies, Carlsbad, CA), 100 ug/mL streptomycin and 100 units/mL penicillin. Cells were incubated at 37C under 95% air/5% CO2 in a standard humidified incubator. (See Supplementary Table 2 for characterization of cells.)

Temperature-sensitive ubiquitination mutant ts20 3T3 cells, derived from the mouse cell line BALB/c 3T3, and NIH 3T3 mouse embryonic fibroblast cells were gifts from H. Ozer [17].

### Immunoblotting analysis and immunopreciptitation assays

These assays were performed as previously described [1]. For proteasome and PKC inhibition, different concentrations of MG132 and bisimdolymaleidmide (Sigma-Aldrich, Saint Louis, Missouri), respectively, were used.

### Split ubiquitin yeast two-hybrid assay

Split ubiquitin yeast two-hybrid assays were performed using a Dual membrane system (Dualsystems Biotech) [13]. Clones were selected on leucine-tryptophan-histidine triple selection plates in the presence of 3mM 3-aminotrazole. Protein interaction was monitored by the expression of the reporter genes *HIS3* and *lacZ,* and the strongest positive clones were identified and used for subsequent studies. The drug resistance profiles were determined by the spot assay [40] by serially diluting overnight grown cultures onto CD complete plates in the absence and presence of valinomycin.

### Ubiquitination and degradation assays

Protein extracts and membrane fractions were prepared as previously described [41]. The turnover of P-gp was assayed by addition of cycloheximide to cultures and subsequent immunoblotting as previously described [42].

*In vivo* degradation of P-gp was performed in the yeast strain expressing P-gp, CD44 and YEp105 which has a c-myc-tagged ubiquitin under the control of *CUP1* promoter. Cultures grown overnight in the selective drop out medium in the presence and absence of CuSO4 were harvested in the mid exponential phase and tested for P-gp degradation [39]. The proteasomal degradation of P-gp was measured both in NIH3T3 cells using a temperature-sensitive proteasome mutant, and in a yeast strain with a conditional *cim5-1* mutant, cotransfected with *MDR1* and *CD44* and its phosphorylation mutants [17, 38]. Cycloheximide chase analysis was carried out on MCF-7/BC19, MCF-7/Adr and in yeast strains.

*In vivo* ubiquitination was studied in HEK293T cells co-transfected with His-tagged *MDR1, CD44* and its phosphorylation defective mutants along with HA-tagged ubiquitin. After 48 hr transfection, the cells were harvested and purified on Ni-NTA resin under denaturing conditions and subsequently analyzed by immunoblotting with anti-HA antibody as described [43]. For *in vitro* ubiquitination assays, recombinant FLAG-P-gp was expressed in insect cells and phosphorylated with protein kinase C (PKC). The baculoviral expression plasmid for SKP1 and the bacterial expression for Cul1-Rbx1 plasmids were gifts from R. Deshaies. Recombinant baculovirus for the expression of P-gp, FBXO21-SKP1 were produced in sf-9 cells using the Baculo Gold Kit (BD). Purified proteins were incubated with E1, UbcH5c, biotinylated ubiquitin and ATP at 37°C for 1 hr followed by SDS-PAGE separation and visualized with a streptavidin HRP conjugate protein detection system (Vector Labs).

### Viability assay

Cell viability was determined by MTT assays following treatment with doxorubicin. P-gp-positive cells transfected with empty vector were used as a control as previously described [44].

### Screening siRNA library

The Silencer siRNA libraries (Ambion) provide 1nmole of each of three individual siRNAs per target for genes covering 325 known and predicted human ubiquitin E3 ligases in 96 well plates. Each siRNA was diluted and aliquoted in black clear-bottom 96-well plates (Corning, 3712) by dispensing 10μl of the corresponding siRNA pools (100nM). 0.5μl of siPORT NeoFXT transfection reagent (Ambion) was diluted in 10μl of Opti-Mem media (Invitrogen) and the mixture was aliquoted to each well and incubated at room temperature for 20 min. MCF-7, MCF-7/P-gp(+) and MCF-7/P-gp(+)/CD44(+) cells were trypsinized, and re-suspended in RPMI medium supplemented with 10% FBS and diluted so that 6000 cells/80μl were delivered to each well. The plates were then incubated at 37°C, in 5% CO2 for 2 days. After 2 days of RNAi treatment, the cells were treated with calcein AM for 30 min. The cells were washed with PBS twice and lysed with 10mM Tris HCl containing 0.2% Triton X100 and the fluorescence at 485/535 nm was measured. Candidate genes displaying a direct effect on the siRNAs only in MCF-7/P-gp(+) and not in MCF-7/P-gp(+)/CD44s(+) or MCF-7/P-gp(−)/CD44(−) cells, with the highest p-value in three independent primary screens were considered as potential hits. The validation of the potential hits was done by transfecting the respective individual siRNAs of each candidate in MCF-7/P-gp(+) and MCF-7/P-gp(+)/CD44s(+) cells and testing for increased drug export (ie, less intracellular calcein AM).

### Plasmids and antibodies

Both full-length *FBXO21* and a deletion mutant construct were cloned into pCMV-Myc-tagged vector. The copper (II) ion inducible ubiquitin expression vector in yeast was a gift from S. Garret. 14E4, a monoclonal antibody raised against a CD44 peptide incorporating a phosphoserine residue at position 325 of CD44 was a gift from C. Isacke. Cul1, Skp1, Roc1 plasmids were a gift from R. Deshaies. FLAG-P-gp was a gift from K. Linton. The retroviral vector pcDNA3.1 (Thermo Fisher Scientific, Waltham, MA) was used to transduce the human CD44s cDNA sequence into MCF-7/BC19 and MCF-7/Adr cells. The EcoR1 digested fragment, which includes the entire CD44s cDNA sequence was treated with T4 DNA polymerase to produce a DNA fragment with blunt ends. The same enzyme was used to fill-in the ends of pcDNA3.1 linearized with Xba1, which was then ligated to the CD44s cDNA insert using T4 DNA ligase. This recombinant plasmid (named pcDNA3.1/CD44s) sequence orientation was confirmed by restriction digestion. pcDNA3.1 was then transfected into the packing cell line PT67 (Clontech) as suggested by the manufacturer. Supernatant from transfected PT67 culture plates was collected from 24 to 72 hours after transfection. About 5 × 10^5^ cells were seeded in 100-mm plates, grown overnight, and infected with virus-containing supernatant from PT67 plates. Single colonies of stable transductants were selected using G-418 for 1 to 2 weeks and transferred to 24-well plates for another 1 to 2 weeks of incubation in the presence of G-418. Cells clones that grew to confluency were transferred to 6-well plates and then to 100-mm plates for the intended experimental use.

### Statistical analysis

Student *t*-test was used for the analysis of the siRNA library screening. We looked for siRNAs that produced a significant reduction in intracellular calcein AM fluorescence uptake on the MCF-7 P-gp(+)/CD44(−) cells, but not on MCF-7 P-gp(−)/CD44(−)or MCF-7 P-gp(+)/CD44(+) cells. To do this, we performed a one-sided Student *t*-test by comparing the mean intracellular fluorescence before and after the siRNA application on the MCF-7 P-gp(+)/CD44(−) cells and filtering based on the criterion that the fluorescence level had to be significantly reduced (*p* < 0.05). We also ensured that the siRNA did not significantly alter the fluorescence level for the parental (MCF-7) and MCF-7 P-gp(+)/CD44(+) cell lines by performing a two-sided *t*-test (*p* > 0.1). From this we obtained a list of candidate siRNAs showing increasing amounts of fluorescence being pumped out of the cell and, therefore, potentially inhibiting P-gp degradation.

## SUPPLEMENTARY MATERIAL FIGURE AND TABLES


