# Supplementary material for: CD44 promotes multi-drug resistance by protecting P-glycoprotein from FBXO21-mediated ubiquitination
Source: Oncotarget. 2015 Jul 3;6(28):26308–21. doi: 10.18632/oncotarget.4763 (PMC4694903; doi:10.18632/oncotarget.4763)
Supplement: Supplementary file 1 [file oncotarget-06-26308-s001.pdf]

CD44 promotes multi-drug resistance by protecting P-glycoprotein from FBXO21-mediated ubiquitination

Supplementary Material

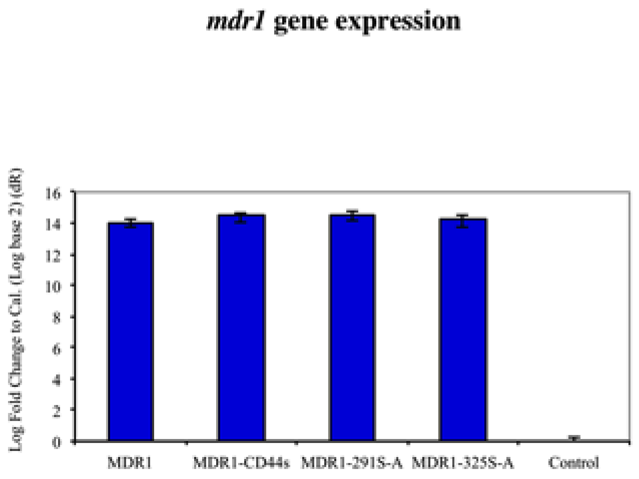

Supplement Figure1. CD44 does not affect the *mdr1* gene expression level. Relative quantification of *mdr1* was performed in yeast strains transformed with *mdr1* alone driven by the TEF1 promoter, or *mdr1* co-transformed with *CD44* or the indicated phosphorylation mutants by Real Time RT-PCR according to the ΔCt comparative method and data are expressed as fold changes compared to the *mdr1* negative wild type yeast strain normalized to the TDH signal.

Screening Results

		P values		
**Gene Name**	**Gene ID**	**MCF-7/Pgp(+)**	**MCF-7/CD44s(+)**	**MCF-7**
MNAT1	4331	1.72E-05	1.12E-01	4.09E-01
FBXO21	23014	2.10E-05	5.49E-01	4.05E-01
TRIM56	81844	1.32E-04	7.82E-01	4.08E-01
LONRF1	164832	1.38E-04	9.87E-01	6.62E-01
TRIM62	55223	4.15E-04	4.82E-01	1.09E-01
RNF115	27246	2.69E-03	1.26E-01	1.07E-01
RNF25	64320	7.83E-03	1.87E-01	5.32E-01
DTX2	113878	1.10E-02	8.20E-01	6.05E-01
FBXL3	26224	1.24E-02	8.00E-01	1.93E-01
CUL5	8065	1.61E-02	7.41E-01	8.97E-01

Supplement Table 1: RNAi screening identifies FBXO21 as a potential ligase candidate for P-gp. Parental MCF-7/(Pgp(-)/CD44(-), MCF-7/Pgp(+)CD44(-), and MCF-7/Pgp(+)CD44(+) cells were screened with an siRNA ubiquitin ligase library targeting 329 known and predicted ubiquitin ligases. Candidate genes displaying a direct effect on calcein accumulation in three independent primary screens are shown.

Cell type	Cell line	P-gp	CD44
Breast cancer	MCF-7	Negative	Negative
	MCF-7/BC19	Positive	Negative
	MCF-7/Adr	Positive	Positive
Ovarian cancer	SKA	Negative	Positive

Supplement Table 2: Cancer cell lines and their respective P-gp and CD44 expression.
